# Controlling Nuclear NF-κB Dynamics by β-TrCP—Insights from a Computational Model

**DOI:** 10.3390/biomedicines7020040

**Published:** 2019-05-27

**Authors:** Uwe Benary, Jana Wolf

**Affiliations:** Mathematical Modelling of Cellular Processes, Max Delbrück Center for Molecular Medicine, 13125 Berlin-Buch, Germany

**Keywords:** mathematical modelling, β-TrCP, NF-κB signaling, drug target, oscillation, fold-change, area under curve, ordinary differential equations

## Abstract

The canonical nuclear factor kappa-light-chain-enhancer of activated B cells (NF-κB) signaling pathway regulates central processes in mammalian cells and plays a fundamental role in the regulation of inflammation and immunity. Aberrant regulation of the activation of the transcription factor NF-κB is associated with severe diseases such as inflammatory bowel disease and arthritis. In the canonical pathway, the inhibitor IκB suppresses NF-κB’s transcriptional activity. NF-κB becomes active upon the degradation of IκB, a process that is, in turn, regulated by the β-transducin repeat-containing protein (β-TrCP). β-TrCP has therefore been proposed as a promising pharmacological target in the development of novel therapeutic approaches to control NF-κB’s activity in diseases. This study explores the extent to which β-TrCP affects the dynamics of nuclear NF-κB using a computational model of canonical NF-κB signaling. The analysis predicts that β-TrCP influences the steady-state concentration of nuclear NF-κB, as well as changes characteristic dynamic properties of nuclear NF-κB, such as fold-change and the duration of its response to pathway stimulation. The results suggest that the modulation of β-TrCP has a high potential to regulate the transcriptional activity of NF-κB.

## 1. Introduction

Nuclear factor kappa-light-chain-enhancer of activated B cells (NF-κB) signaling is involved in key cellular processes, such as proliferation, differentiation, and apoptosis, and plays a fundamental role in the regulation of inflammation and immunity [1,2]. NF-κB signaling is traditionally divided into two main pathway branches: The canonical and the non-canonical signaling pathway [3,4]. Here, we focus on the canonical pathway, in which the activity of the transcription factor NF-κB (p50/p65) is regulated by the abundance of the inhibitor IκBα, which sequesters NF-κB in the cytoplasm. If extracellular TNFα stimulates a cell, this signal is transduced intracellularly via the canonical pathway, resulting in the activation of the IκB kinase complex (IKK). Activated IKK phosphorylates IκB, which allows for the ubiquitination of IκB through a mechanism mediated by β-TrCP [4,5,6]. Ubiquitinated IκB is degraded by the proteasome, and, consequently, NF-κB is released from its complex with IκB. NF-κB can now translocate to the nucleus and regulate the transcription of target genes.

Aberrations in the regulation of NF-κB activation are associated with severe diseases such as arthritis, Crohn’s disease, and autoimmune diseases. The underlying molecular mechanisms have not been clarified in all cases, since mutations are rarely found in components of this pathway [2,7]. Up-regulated NF-κB activity has been detected in various human cancers, potentially inducing the secretion of tumor-promoting cytokines and promoting cell resistance to anti-cancer therapies [2,8,9,10,11,12,13]. These observations have made the molecular processes that regulate NF-κB activity, especially the mechanisms involved in proteasomal IκB degradation, targets for the development of therapeutic approaches [2,3,14]. For instance, IKK inhibitors have been developed in hopes of achieving anti-tumor and anti-inflammatory effects, but they have had only limited success so far [2,3,5,11]. Strategies of targeting the proteasome itself, with Bortezomib and other inhibitors, have been used in clinics, but such inhibitors are less specific and thus cause undesirable side effects [2,5,10,11,15,16]. Reducing side effects will likely require a more targeted approach within the NF-κB pathway [3,5,11,17].

β-TrCP’s specificity as the central mediator of IκB degradation has attracted interest as promising pharmacological target that should be accompanied by fewer side effects [3,5,11,15,17,18]. Two paralogues of β-TrCP, β-TrCP1/FWD1 and β-TrCP2/HOS, exist in mammals. The paralogues are highly conserved within their functionally important F-box domain and β-transducin repeats but differ in their N-termini [19,20,21]. Generally, β-TrCP1/FWD1 and β-TrCP2/HOS are considered to be functionally redundant in the NF-κB signaling pathway [5,9,15,17,22]. This generally accepted notion of redundancy of the paralogues has been inferred from (i) their high degree of 77% sequence homology [21], (ii) their identical biochemical properties in vitro [15], (iii) their apparent reciprocal substitution in paralogue-specific small interfering RNA (siRNA)-mediated knock-down experiments [23], and (iv) the general viability of β-TrCP1/FWD1 knock-out mice [15,23,24]. 

In addition to IκB, β-TrCP targets several other substrates for proteasomal degradation, such as Cdc25A, ATF, Per, and β-catenin [15,17]. In addition, several signaling pathways and regulatory molecules were shown to modulate the abundance of β-TrCP, including Wnt/β-catenin signaling [17,25,26], BMP/MAPK [27], Ras/MAPK [28], Src [29], JNK and Akt/PKB signaling [25,30], Ras/NORE1A [31], Vpu [32], and TRIM9 [22]. Thus, β-TrCP is in the center of a complex interaction network. We focus our study on β-TrCP’s influence on NF-κB signaling and leave potential effects on the network to the discussion section.

In our study, we explore what impact the modulation of β-TrCP-mediated IκB degradation has on the dynamics of nuclear NF-κB. To do so, we make use of a computational approach. Many computational models have been published to describe NF-κB signaling in order to investigate different biological questions [33,34,35,36,37,38,39,40,41,42,43,44,45,46,47], including the prediction of drug effects [48,49,50,51]. Despite the molecular elaborateness of several of these models, β-TrCP does not appear in any of them. We extend the detailed model of canonical NF-κB signaling established by Lipniacki et al. [34] by integrating β-TrCP-mediated IκB degradation and carry out a comprehensive analysis of β-TrCP’s regulatory impact on nuclear NF-κB concentration.

## 2. Materials and Methods

### 2.1. Modelling Canonical NF-κB Signaling

**Figure 1:**
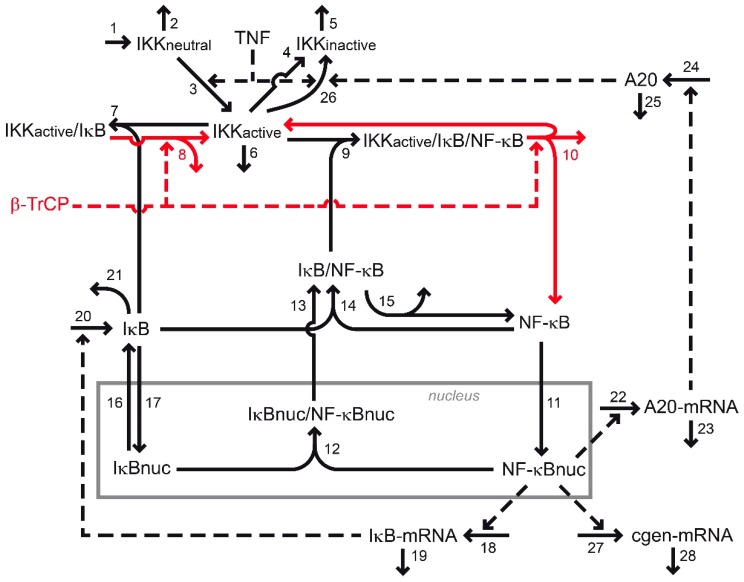
Reaction scheme underlying the model of canonical NF-κB signaling. Reactions and pathway components of the original model [34] are shown in black; reactions involving β-TrCP-mediated IκB degradation are highlighted in red indicating model extensions. The nuclear species of the model are surrounded by the grey box. The numbers next to the arrows specify the number of the reaction. One-headed arrows denote reactions taking place in the indicated direction. Dashed arrows illustrate activations. Components in a complex are separated by slashes. The Methods Section provides additional explanations; Document S1 lists reaction rates and parameters of the model. cgen: Control gene; nuc: Nuclear.

The published model of the NF-κB pathway [34] consists of 14 ordinary differential equations (ODEs) and one conservation relation for NF-κB (Document S1). The model includes three activation forms of the IKK complex: A neutral form (IKKneutral), an active form (IKKactive), and an inactive form (IKKinactive). All three types are degraded (reaction 2, reaction 5, and reaction 6 in Figure 1), but only IKKneutral is produced de novo (reaction 1). TNF stimulation activates IKKneutral (reaction 3). TNF is implemented into the model as a logical variable that is either set to 0 in the absence of a stimulus or to 1 if a stimulus is present (Document S1). Besides IKKneutral activation, TNF also promotes the inactivation of IKKactive (reaction 26). In addition, IKKactive inactivates itself in a TNF-independent manner (reaction 4). IKKactive binds NF-κB-bound and unbound IκB (reaction 7 and reaction 9, respectively). IκB is degraded in an IKKactive-dependent (reaction 8 and reaction 10) and IKKactive-independent manner (reaction 15 and reaction 21). The IKKactive-dependent degradation of IκB via reaction 10 results in the dissociation of the IKKactive/IκB/NF-κB complex, liberating NF-κB from its inhibitor. NF-κB can then translocate into the nucleus (reaction 11). In the nucleus, NF-κB regulates the transcription of IκB-mRNA, A20-mRNA, and the control gene transcript “cgen-mRNA” (reaction 18, reaction 22, and reaction 27, respectively). IκB-mRNA, A20-mRNA, and cgen-mRNA are degraded via reaction 19, reaction 23, and reaction 28, respectively. IκB-mRNA and A20-mRNA are furthermore translated into their respective proteins (reaction 20 and reaction 24, respectively). A20 proteins promote the inactivation of IKKactive (reaction 26) and are degraded via reaction 25. IκB can either associate with NF-κB in the cytoplasm to form IκB/NF-κB complexes (reaction 14) or shuttle between the cytosolic and nuclear compartment (reaction 16 and reaction 17). Nuclear IκB (IκBnuc) associates with nuclear NF-κB (NF-κBnuc) to form nuclear IκBnuc/NF-κBnuc complexes (reaction 12). These nuclear complexes translocate from the nuclear to the cytosolic compartment (reaction 13).

The model is used to analyze the impact of the regulation of β-TrCP-mediated IκB degradation on the dynamical properties of the nuclear NF-κB response to TNF stimulation. To this end, the model was extended such that the IKKactive-dependent degradation of IκB was regulated by β-TrCP (reaction 8 and reaction 10).

### 2.2. Measures of Nuclear NF-κB Dynamics

To quantitatively characterize the dynamics of species in ODE models, several measures have been introduced, reviewed in [52]. Here, we focused on two measures, signal duration and fold-change [26,53,54,55,56], and defined them as follows:

(1) $$\mathrm{signal}\;\mathrm{duration}=\sqrt{\frac{\int_{t_{initial}}^{t_{final}}t^{2}\cdot \left|\frac{d(\mathrm{NF}-\kappa B)\left[t\right]}{dt}\right|dt}{\int_{t_{initial}}^{t_{final}}\left|\frac{d(\mathrm{NF}-\kappa B)\left[t\right]}{dt}\right|dt}-{\left(\frac{\int_{t_{initial}}^{t_{final}}t\cdot \left|\frac{d(\mathrm{NF}-\kappa B)\left[t\right]}{dt}\right|dt}{\int_{t_{initial}}^{t_{final}}\left|\frac{d(\mathrm{NF}-\kappa B)\left[t\right]}{dt}\right|dt}\right)}^{2}}$$

(2) $$\mathrm{fold}-\mathrm{change}=\frac{\max\left((\mathrm{NF}-\kappa B)\left[t\right]\right)}{(\mathrm{NF}-\kappa B)\left[t_{initial}\right]}\;\mathrm{with}\;t_{initial}\leq t\leq t_{final}$$

These definitions can be applied to the wide range of different dynamical responses [26,53,54,55]. They do not require that the signal eventually returns to its initial steady state, and they are also suitable for oscillatory transitions. Numerically calculated steady states were used as initial conditions to simulate the continuous dynamics of model species over time to the final time point of 630 min. 

### 2.3. Bifurcation Analysis

We performed a bifurcation analysis by numerically calculating all steady states and their stability for distinct values of the bifurcation parameter β-TrCP, as described in [26] using Mathematica 10.0 (Wolfram Research, Champaign, IL, USA). Briefly, to calculate steady states, we set all time derivatives of the model to zero and solved the resulting system of algebraic equations for species concentrations, considering only non-negative real solutions. We determined steady-state stability by calculating the eigenvalues of the Jacobian matrix [57]. The Jacobian matrix is the matrix of all first-order partial derivatives of the ODEs, with respect to the species of the model. If the real parts of all eigenvalues are negative, the steady state is called stable. Otherwise, the steady state is called unstable. A Hopf bifurcation is detected if all eigenvalues of the Jacobian matrix have negative real parts, with the exception of one conjugate purely imaginary pair. The period length of oscillations is calculated by identifying the maximal frequency in the discrete Fourier transform of a simulated time course of nuclear NF-κB.

## 3. Results

### 3.1. Description of the Computational Model of Canonical NF-κB Signaling

Our starting point to model NF-κB signaling was the detailed kinetic model published by Lipniacki and colleagues [34]. This model quantitatively describes the molecular processes that transduce an extracellular TNF signal into a change in nuclear NF-κB concentrations. The model accounts for transient TNF-dependent IKK activation, NF-κB-regulated target gene expression (cgen-mRNA), and the inhibitory actions of IκB and A20 on NF-κB activation (Figure 1). The parameters used in the model have either been measured or good estimates have been derived to quantitatively describe the temporal changes observed in concentrations of pathway components in experiments [34].

The original model does not incorporate β-TrCP explicitly. To allow for the investigation of the potential influence of β-TrCP on nuclear NF-κB dynamics, we extended the existing model based on published experimental data. Experimental observations suggest that changes in the concentration of β-TrCP influence IκB degradation. The overexpression of β-TrCP reduced the concentration of IκB by enhancing its proteasomal degradation [19,21,58,59,60]. In contrast, the overexpression of a dominant negative mutant of β-TrCP inhibited the proteasomal degradation of IκB [19,21,58,59,60]. Furthermore, it was shown that the IKK-dependent phosphorylation of IκB is a prerequisite for the β-TrCP-mediated ubiquitination and degradation of IκB. Thus, we presumed β-TrCP to modulate the rates of both IKK-dependent IκB degradation reactions (reaction 8 and reaction 10, Figure 1) in our modelling approach.

**Figure 2:**
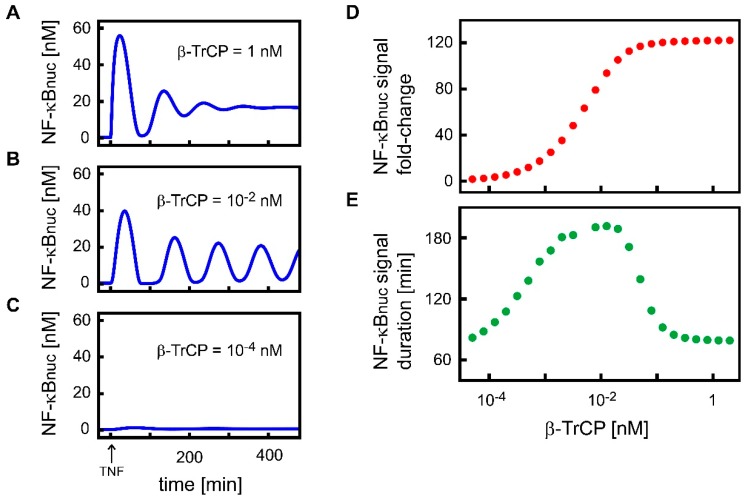
Impact of β-TrCP abundance on the transient dynamics of nuclear NF-κB upon TNF stimulation. (**A**–**C**) Simulations of the dynamics of nuclear NF-κB (NF-κBnuc) upon TNF stimulation are shown assuming (**A**) 1 nM β-TrCP, (**B**) 10^−2^ nM β-TrCP, and (**C**) 10^−4^ nM β-TrCP. (**D**) Dependence of the fold-change of nuclear NF-κB upon TNF stimulation assuming β-TrCP concentrations between 5 × 10^−5^ and 2 nM. (**E**) Influence of β-TrCP abundance on signal duration of nuclear NF-κB dynamics upon TNF stimulation assuming β-TrCP concentrations between 5 × 10^−5^ and 2 nM.

β-TrCP is thought to be expressed at low levels in cells [17], although absolute concentrations have not yet been published. Consequently, we varied the concentration of β-TrCP over a wide range in our model analyses to cover all possible conditions. The β-TrCP concentration of 1 nM is a special case. In that situation, the extended model and the original model published by Lipniacki and colleagues behave identically. Figure 2A shows the transient dynamics of nuclear NF-κB (NF-κBnuc) upon TNF stimulation, assuming a β-TrCP concentration of 1 nM. Nuclear NF-κB dynamics was, in this case, characterized by a transient increase in the nuclear NF-κB concentration to about 56 nM in the first 90 min after TNF stimulation (first peak), followed by four minor peaks with decreasing amplitudes indicating damped oscillations.

### 3.2. β-TrCP Abundance Influences the Transient Dynamics of Nuclear NF-κB upon TNF Stimulation

Simulations (Figure 2A–C, Model S1) showed that the dynamics of nuclear NF-κB upon TNF stimulation differ when concentrations of β-TrCP change. Choosing values of 10^−4^, 10^−2^, and 1 nM (Figure 2A–C) showed that higher β-TrCP concentrations led to larger maxima of the first peak of nuclear NF-κB concentration (1.4, 40, and 56 nM, respectively). The maxima of the consecutive peaks were also affected.

Different features of nuclear NF-κB dynamics have been proposed to correlate with the responses of NF-κB target genes on expression levels [37,61], such as the magnitude of nuclear NF-κB [56,62,63,64], the number of peaks (duration of oscillations) [62,63], and the inter-peak intervals (period) [46,65]. To quantify these features, different measures have been proposed, such as the amplitude of the first peak [62,63,64,66,67], maximal fold-change [43,56,68,69], the accumulative response (area under curve) [48,56,67,68,69], steady state concentration upon TNF stimulation [66], the timing of the first peak [62,63,64,66,67], length of inter-peak intervals [62,65,66], and duration of the oscillations [62,63,66]. Here, we focused on two established measures, fold-change and signal duration, which are defined in the Methods Section. Fold-change can be interpreted as a measure of the magnitude of NF-κB activation, while signal duration characterizes temporal aspects of the NF-κB response to TNF stimulation.

To analyze the influence of β-TrCP abundance on the dynamics of nuclear NF-κB, we systematically calculated the fold-change of nuclear NF-κB dynamics upon TNF stimulation, assuming β-TrCP concentrations between 5 × 10^−5^ and 2 nM (Figure 2D). The simulations demonstrated that increasing β-TrCP concentrations increased the fold-change of nuclear NF-κB from approximately 1 to 122 fold (Figure 2D). This analysis indicates that β-TrCP concentrations below a critical level (about 10^−4^ nM) impede the response of nuclear NF-κB to TNF stimulation (e.g., compare with Figure 2C).

Calculations showed that the signal duration of nuclear NF-κB upon TNF stimulation also depends on β-TrCP concentration, but, in this case, the relationship was biphasic (Figure 2E). Concentrations of β-TrCP up to approximately 0.01 nM caused the signal duration to rise from about 80 to 190 min. Beyond that critical β-TrCP concentration, however, the signal duration dropped to about 80 min again (Figure 2E). Simulations indicated that this change in signal duration reflects a change in the dampening of nuclear NF-κB oscillations (Figure 2A–C). The closer the β-TrCP concentration approached the value of 0.01 nM, the less dampened nuclear NF-κB dynamics was and, as a result, the longer the NF-κB response to TNF stimulation lasted.

We additionally analyzed β-TrCP’s influence on the area under the curve, signal amplitude (amplitude of 1st peak), and signaling time, which represent three additional established measures (Figure S1). We found that the area under the curve and signal amplitude increased with increasing concentrations of β-TrCP, similar to fold-change. Signaling time showed a biphasic relation with β-TrCP, similar to signal duration.

To summarize, β-TrCP abundance affects all investigated measures—the fold-change and duration of the nuclear NF-κB signal, in particular. The analysis also indicates that a critical minimal β-TrCP concentration is necessary to observe a response of nuclear NF-κB to TNF stimulation and that intermediate β-TrCP concentrations of about 0.01 nM prolong NF-κB oscillations.

### 3.3. β-TrCP Abundance Affects Long-Term Dynamical Behavior of Nuclear NF-κB

**Figure 3:**
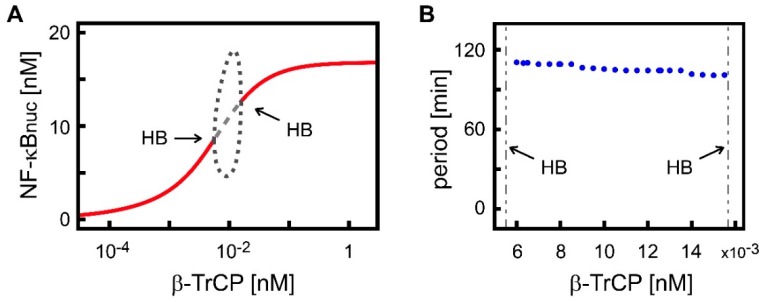
Bifurcation analysis of the stimulated steady state of nuclear NF-κB with respect to β-TrCP abundance. (**A**) β-TrCP abundance influences the stimulated steady-state concentration of nuclear NF-κB and the stability of this steady state. The thick red lines denote stable stimulated steady states of nuclear NF-κB (NF-κBnuc). The grey dashed line denotes unstable stimulated steady states. The dotted lines show minimal and maximal values of the amplitude of stable limit cycle oscillations occurring between the two Hopf bifurcation (HB) points at approximately 5.5 × 10^−3^ and 1.6 × 10^−2^ nM. (**B**) Calculated period length of the limit cycle oscillations simulated for selected values of β-TrCP concentrations between the Hopf bifurcation points (indicated by dashed grey lines). The period hardly changes for the considered values of β-TrCP.

The previous analysis showed that the abundance of β-TrCP influences several features that are characteristic of the transient dynamics of nuclear NF-κB after cellular stimulation by TNF. Next, we investigated its influence on long-term dynamics. Figure 3A shows how long-term dynamics are affected by concentrations of β-TrCP between 5·× 10^−5^ and 2 nM. Raising the concentration of β-TrCP increased the steady-state concentration of nuclear NF-κB reached after TNF stimulation. The more β-TrCP that was available, the more IκB was ubiquitinated and degraded, which resulted in the release of NF-κB from IκB/NF-κB complexes and accumulation of NF-κB in the nucleus. This accumulation had an upper limit of about 16.7 nM, which was reached at β-TrCP concentrations of about 1 nM; higher β-TrCP levels led to very little further change in nuclear NF-κB (Figure 3A). Below a certain concentration (about 10^−4^ nM), concentrations of β-TrCP also had very little impact on the stimulated steady-state concentration of nuclear NF-κB. In these cases, the stimulated steady-state concentration was approximately the same as the steady-state concentration of nuclear NF-κB in the absence of TNF stimulation (about 0.46 nM).

The abundance of β-TrCP also influences the stability of the stimulated steady state. A bifurcation analysis revealed two Hopf bifurcation (HB) points, at approximately 5.5 × 10^−3^ and 1.6 × 10^−2^ nM (Figure 3A). Between these two HB points, the stimulated steady state was unstable (Figure 3A; dashed line) and stable limit cycle oscillations of nuclear NF-κB existed. The oscillations had period lengths within a narrow range, lasting from 100 to 112 min, depending on the value of β-TrCP concentration (Figure 3B). The minimal and maximal values of the amplitude of nuclear NF-κB oscillations, however, varied widely for β-TrCP concentrations between the two HB points (Figure 3A; dotted lines).

These results show that the regulation of β-TrCP abundance can affect both the stimulated steady-state concentration of nuclear NF-κB and its stability. Therefore, the abundance of β-TrCP potentially determines whether damped oscillations or sustained limit-cycle oscillations are observed upon TNF stimulation.

## 4. Discussion

This investigation was motivated by recent interest in the molecule β-TrCP as a potential therapeutic target in modulating cellular signal transduction involving NF-κB. Aberrations in NF-κB signaling are well documented in many types of cancer and other diseases, and a number of approaches have been developed to target components of the pathway. Finding a way to control the concentration of NF-κB in the nucleus has been central to this idea. Attempts to target regulators of NF-κB have led to advances in clinical treatment, e.g., using Bortezomib, but were limited in their success due to non-specific effects—a situation that has stimulated interest in pathway components such as β-TrCP [3,5,11,17]. Our aim was to explore and quantify to what extent β-TrCP could regulate nuclear NF-κB.

We demonstrated that the regulation of β-TrCP-mediated IκB degradation can affect the steady-state concentration of nuclear NF-κB, as well as many characteristics of the dynamics of nuclear NF-κB upon TNF stimulation (Figure 2, Figure 3, Figure S1). This is in accordance with the general opinion that the control of β-TrCP expression level is a very important factor in the regulation of NF-κB signaling [5,17,19,25]. Our model analysis predicts that enhancing the β-TrCP-mediated degradation of IκB increases the steady-state concentration of nuclear NF-κB (Figure 3A). This prediction agrees with many experimental observations based on the overexpression of β-TrCP in various mammalian cell types [8,19,21,25,30,58,60]. It also corroborates the hypothesis that upregulated β-TrCP, which is frequently observed in various human cancers, supports tumorigenesis by activating NF-κB-dependent anti-apoptotic pathways [5,8,11,17]. Our model analysis further predicts that β-TrCP abundance affects transient nuclear NF-κB dynamics in response to TNF stimulation of the pathway. Our results were confirmed in two other NF-κB pathway models [43,65] that differ in their model structure and parametrization from our model (Figure S2), demonstrating the robustness of our predictions.

Negative feedback mechanisms, such as those which occur via IκB and A20, can create oscillations in the dynamics of the pathway components [70,71,72]. Nuclear NF-κB concentrations, for example, have been observed to exhibit sequences of peaks when cells are stimulated by TNFα under certain experimental conditions [33,62,63,65,73,74,75]. Whether nuclear NF-κB exhibits oscillations under all cellular conditions is a subject of ongoing investigation, as is their detailed role in the control of the expression of target genes and their subsequent physiological effects [43,46,62,63,73,76,77,78,79,80]. One proposal has been that the number, period, and/or amplitude of oscillation peaks determine the functional consequences of NF-κB signaling [46,62,63,79,80,81]. The cited biological literature usually defines oscillations as consecutive sequences of three or more concentration peaks. Depending on the total number of observed peaks, the oscillations may be additionally categorized into damped or sustained oscillations. However, from the data that is available, it is difficult to draw conclusions about the existence of sustained oscillations (i.e., limit cycle oscillations in mathematical terms). Our analysis with respect to β-TrCP revealed two Hopf bifurcation points, implying that β-TrCP abundance may determine whether oscillations of the nuclear NF-κB concentration that occur upon TNF stimulation are damped or sustained. The period length of the simulated limit cycle oscillations remained almost constant at approximately 100 to 110 min for different β-TrCP concentrations (Figure 3B), which is in agreement with inter-peak intervals that have been experimentally measured in mouse fibroblasts [33,62,63,73,74,75] and human cells [46,78]. While this period length remains almost constant, the amplitude of the limit cycle oscillations is strongly dependent on the concentrations of β-TrCP (Figure 3A). We thus conclude that β-TrCP abundance influences all of the measures of nuclear NF-κB dynamics that are under discussion with the exception of the length of the oscillation period, and that, in consequence, this probably has an influence on the expression of NF-κB target genes.

Our analyses predict that modulating β-TrCP-dependent IκB degradation would have a strong regulatory impact on the dynamics of nuclear NF-κB for a broad range of cellular β-TrCP concentrations. However, potential drugs will likely change β-TrCP’s activity only transiently depending on their pharmacokinetic properties—a point we have not yet addressed. We thus simulated different possible kinetic profiles of drug action and showed that the response dynamics of nuclear NF-κB upon TNF stimulation can change in the presence of the drug (Figure S3A–E). The extent of the drug’s effect depends on the particular specifics of the pharmacokinetics as well as the concentration of β-TrCP.

Whether β-TrCP could represent a useful pharmacological target has been discussed at several occasions [3,5,11,15,17,18]. A central counter-argument against such an approach has been that β-TrCP recognizes several other signaling molecules in addition to IκB. The regulation of multiple substrates by β-TrCP suggests that targeting β-TrCP could cause undesirable side effects outside of NF-κB signaling. An example is β-catenin, the transcriptional regulator in Wnt/β-catenin signaling [12,13,15,17,82,83]. β-Catenin was shown to directly bind to the p65 and p50 subunits of NF-κB [84,85,86,87]. In this way, Wnt/β-Catenin signaling may create additional complexity to the regulation of NF-κB dynamics by β-TrCP. To investigate this mechanism of regulation, a computational model of crosstalk between NF-κB and Wnt/β-catenin signaling is needed. 

We argue here that targeting β-TrCP may still be sensible despite its multiple substrates. Essentially, β-TrCP’s activity can be changed by the control of its availability for its substrate, i.e., its abundance, and/or by the modulation of its physicochemical properties, i.e., association and dissociation rates of β-TrCP and its substrate. We argue that if a potential drug does not affect β-TrCP’s abundance but does affect its binding to IκB, it may be feasible to achieve NF-κB pathway specificity. To support this idea, we simulated a kinetic profile of drug action, assuming that the drug inhibits the activity of β-TrCP, by reducing the rate of IκB binding. The simulations demonstrate that the influence of the drug on nuclear NF-κB dynamics (Figure S3F) is identical to that of a drug, which modulates β-TrCP’s abundance (Figure S3B(ii)). Importantly, the drug that inhibits IκB binding does not change β-TrCP’s abundance and may consequently preclude effects on other β-TrCP substrates. Small molecules that affect the association of β-TrCP with IκB have already been identified and are being used in experimental settings, including the small molecule inhibitor of IκBα ubiquitination (GS143) [88,89]. GS143 appears to be specific to the NF-κB pathway [88,89]; at least, it does not simultaneously promote the activation of Wnt/β-catenin signaling.

In summary, the results from our computational model analyses confirm that targeting β-TrCP has a great potential to regulate nuclear NF-κB responses in various ways. We conclude that the modulation of β-TrCP is a useful tool to modify pathway dynamics and offers a productive strategy to investigate their impact on target gene expression. How this principle can be applied in clinical treatments will depend on further research to determine which characteristics of the nuclear NF-κB dynamics are most crucial in a particular pathophysiological condition.

## Supplementary Materials

The following are available online at https://www.mdpi.com/2227-9059/7/2/40/s1. Document S1: Document_S1.doc provides the detailed description of the modelling approach listing equations and parameters. Figure S1: Figure_S1.pdf shows the influence of β-TrCP on additional measures of NF-κB dynamics. Figure S2: Figure_S2.pdf provides confirmation of our results in two other NF-κB pathway models. Figure S3: Figure_S3.pdf shows the influence of a kinetic profile of a drug targeting β-TrCP. Model S1: The Mathematica notebook NFkB-Model_UBenary_etal.nb provides the ready-to-use code to reproduce Figure 2A. The code can be easily extended to reproduce any figures and data of this manuscript.
