# Supplementary material for: Controlling Nuclear NF-κB Dynamics by β-TrCP—Insights from a Computational Model
Source: Biomedicines. 2019 May 27;7(2):40. doi: 10.3390/biomedicines7020040 (PMC6631534; doi:10.3390/biomedicines7020040)
Supplement: Supplementary file 1 [file biomedicines-07-00040-s001.zip › Document_S1.pdf]

Supplemental Document S1

Controling Nuclear NF-κB Dynamics by β-TrCP—Insights from a Computational Model.

Uwe Benary & Jana Wolf

Table of contents

1. The detailed kinetic model of canonical NF-κB signalling 1

1.1 Differential equations 2
1.2 Conservation relation 3
1.3 Rate equations 3
1.4 Stimulation by TNF 4
1.5 Model parameters 5

2. References 6

1. The detailed kinetic model of canonical NF-κB signalling

A schematic representation of the detailed kinetic model of canonical NF-κB signalling is shown in Figure 1. In the scheme, the number next to an arrow denotes the number of the particular reaction. The rate equations that describe these reactions are provided in Section 1.3. In the rate equations, components in a complex are separated by a slash. The model is given by the Equations 1.1 - 1.44. The parameters are listed in Table S1.

1.1 Differential equations

$$\frac{d(IKKn)}{dt} = v_1 - v_2 - v_3 \quad 1.1$$

$$\frac{d(IKKi)}{dt} = v_4 - v_5 + v_{26} \quad 1.2$$

$$\frac{d(IKKa)}{dt} = v_3 - v_4 - v_6 - v_7 + v_8 - v_9 + v_{10} - v_{26} \quad 1.3$$

$$\frac{d(IKKa/I\kappa B)}{dt} = v_7 - v_8 \quad 1.4$$

$$\frac{d(IKKa/I\kappa B/NF\text{-}\kappa B)}{dt} = v_9 - v_{10} \quad 1.5$$

$$\frac{d(I\kappa B)}{dt} = -v_7 - v_{14} + v_{16} - v_{17} + v_{20} - v_{21} \quad 1.6$$

$$\frac{d(NF\text{-}\kappa B)}{dt} = v_{10} - v_{11} - v_{14} + v_{15} \quad 1.7$$

$$\frac{d(I\kappa Bnuc)}{dt} = -v_{12} - k_v \cdot v_{16} + k_v \cdot v_{17} \quad 1.8$$

$$\frac{d(NF\text{-}\kappa Bnuc)}{dt} = k_v \cdot v_{11} - v_{12} \quad 1.9$$

$$\frac{d(I\kappa Bnuc/NF\text{-}\kappa Bnuc)}{dt} = v_{12} - k_v \cdot v_{13} \quad 1.10$$

$$\frac{d(I\kappa B\text{-}mRNA)}{dt} = v_{18} - v_{19} \quad 1.11$$

$$\frac{d(A20\text{-}mRNA)}{dt} = v_{22} - v_{23} \quad 1.12$$

$$\frac{d(A20)}{dt} = v_{24} - v_{25} \quad 1.13$$

$$\frac{d(cgen\text{-}mRNA)}{dt} = v_{27} - v_{28} \quad 1.14$$

1.2 Conservation relation

$$(NF\text{-}\kappa B)_{total} = (IKKa/I\kappa B/NF\text{-}\kappa B) + (NF\text{-}\kappa B) + (I\kappa B/NF\text{-}\kappa B) + \frac{1}{k_v} \cdot (NF\text{-}\kappa Bnuc)$$

$$+ \frac{1}{k_v} \cdot (I\kappa Bnuc/NF\text{-}\kappa Bnuc) \quad 1.15$$

1.3 Rate equations

$$v_1 = constant \quad 1.16$$

$$v_2 = k_2 \cdot (IKKn) \quad 1.17$$

$$v_3 = k_3 \cdot TNF \cdot (IKKn) \quad 1.18$$

$$v_4 = k_4 \cdot (IKKa) \quad 1.19$$

$$v_5 = k_5 \cdot (IKKi) \quad 1.20$$

$$v_6 = k_6 \cdot (IKKa) \quad 1.21$$

$$v_7 = k_7 \cdot (IKKa) \cdot (I\kappa B) \quad 1.22$$

$$v_8 = k_8 \cdot (\beta\text{-}TrCP) \cdot (IKKa/I\kappa B) \quad 1.23$$

$$v_9 = k_9 \cdot (IKKa) \cdot (I\kappa B/NF\text{-}\kappa B) \quad 1.24$$

$$v_{10} = k_{10} \cdot (\beta\text{-}TrCP) \cdot (IKKa/I\kappa B/NF\text{-}\kappa B) \quad 1.25$$

$$v_{11} = k_{11} \cdot (NF\text{-}\kappa B) \quad 1.26$$

$$v_{12} = k_{12} \cdot (I\kappa Bnuc) \cdot (NF\text{-}\kappa Bnuc) \quad 1.27$$

$$v_{13} = k_{13} \cdot (I\kappa Bnuc/NF\text{-}\kappa Bnuc) \quad 1.28$$

$$v_{14} = k_{14} \cdot (I\kappa B) \cdot (NF\text{-}\kappa B) \quad 1.29$$

$$v_{15} = k_{15} \cdot (I\kappa B/NF\text{-}\kappa B) \quad 1.30$$

$$v_{16} = k_{16} \cdot (I\kappa Bnuc) \quad 1.31$$

$$v_{17} = k_{17} \cdot (I\kappa B) \quad 1.32$$

$$v_{18} = k_{18} \cdot (NF\text{-}\kappa Bnuc) \quad 1.33$$

$$v_{19} = k_{19} \cdot (I\kappa B\text{-}mRNA) \quad 1.34$$

$$v_{20} = k_{20} \cdot (I\kappa B\text{-}mRNA) \quad 1.35$$

$$v_{21} = k_{21} \cdot (I\kappa B) \quad 1.36$$

$$v_{22} = k_{22} \cdot (NF\text{-}\kappa Bnuc) \quad 1.37$$

$$v_{23} = k_{23} \cdot (A20\text{-}mRNA) \quad 1.38$$

$$v_{24} = k_{24} \cdot (A20\text{-}mRNA) \quad 1.39$$

$$v_{25} = k_{25} \cdot (A20) \quad 1.40$$

$$v_{26} = k_{26} \cdot TNF \cdot (IKKa) \cdot (A20) \quad 1.41$$

$$v_{27} = k_{27} \cdot (NF\text{-}\kappa Bnuc) \quad 1.42$$

$$v_{28} = k_{28} \cdot (cgen\text{-}mRNA) \quad 1.43$$

1.4 Stimulation by TNF

The TNF stimulus is simulated by incrementing TNF in Equation 1.18 and Equation 1.41 from 0 to 1 at the time point of stimulus application.

$$TNF_{t=0} = 0 \text{ and } TNF_{t>0} = 1 \quad 1.44$$

1.5 Model parameters

Table S1: Parameters of the detailed kinetic model of canonical NF-κB signalling.
The parameter values are adopted from [1].

Parameter	Value	
v_1	1.5	$nM \cdot min^{-1}$
k_2	$7.5 \cdot 10^{-3}$	min^{-1}
k_3	0.15	min^{-1}
k_4	$9 \cdot 10^{-2}$	min^{-1}
k_5	$7.5 \cdot 10^{-3}$	min^{-1}
k_6	$7.5 \cdot 10^{-3}$	min^{-1}
k_7	$1.2 \cdot 10^{-2}$	$nM^{-1} \cdot min^{-1}$
k_8	6	$nM^{-1} \cdot min^{-1}$
k_9	$6 \cdot 10^{-2}$	$nM^{-1} \cdot min^{-1}$
k_{10}	6	$nM^{-1} \cdot min^{-1}$
k_{11}	0.15	min^{-1}
k_{12}	$3 \cdot 10^{-2}$	$nM^{-1} \cdot min^{-1}$
k_{13}	0.6	min^{-1}
k_{14}	$3 \cdot 10^{-2}$	$nM^{-1} \cdot min^{-1}$
k_{15}	$1.2 \cdot 10^{-3}$	min^{-1}
k_{16}	$3 \cdot 10^{-2}$	min^{-1}
k_{17}	$6 \cdot 10^{-2}$	min^{-1}
k_{18}	$3 \cdot 10^{-5}$	min^{-1}

Table S1 continues at the next page.

Table S1: Parameters of the detailed kinetic model of canonical NF-κB signalling. (continued)

Parameter	Value	
k_{19}	$2.4 \cdot 10^{-2}$	min^{-1}
k_{20}	30	min^{-1}
k_{21}	$6 \cdot 10^{-3}$	min^{-1}
k_{22}	$3 \cdot 10^{-5}$	min^{-1}
k_{23}	$2.4 \cdot 10^{-2}$	min^{-1}
k_{24}	30	min^{-1}
k_{25}	$1.8 \cdot 10^{-2}$	min^{-1}
k_{26}	$6 \cdot 10^{-3}$	$nM^{-1} \cdot min^{-1}$
k_{27}	$3 \cdot 10^{-5}$	min^{-1}
k_{28}	$2.4 \cdot 10^{-2}$	min^{-1}
$(NF\text{-}\kappa B)_{total}$	60	nM
k_v	5	

2. References

1. Lipniacki, T., et al., *Mathematical model of NF-kappaB regulatory module.* J Theor Biol, 2004. **228**(2): p. 195-215.
