# Supplementary material for: Controlling Nuclear NF-κB Dynamics by β-TrCP—Insights from a Computational Model
Source: Biomedicines. 2019 May 27;7(2):40. doi: 10.3390/biomedicines7020040 (PMC6631534; doi:10.3390/biomedicines7020040)
Supplement: Supplementary file 1 [file biomedicines-07-00040-s001.zip › Figure_S1.pdf]

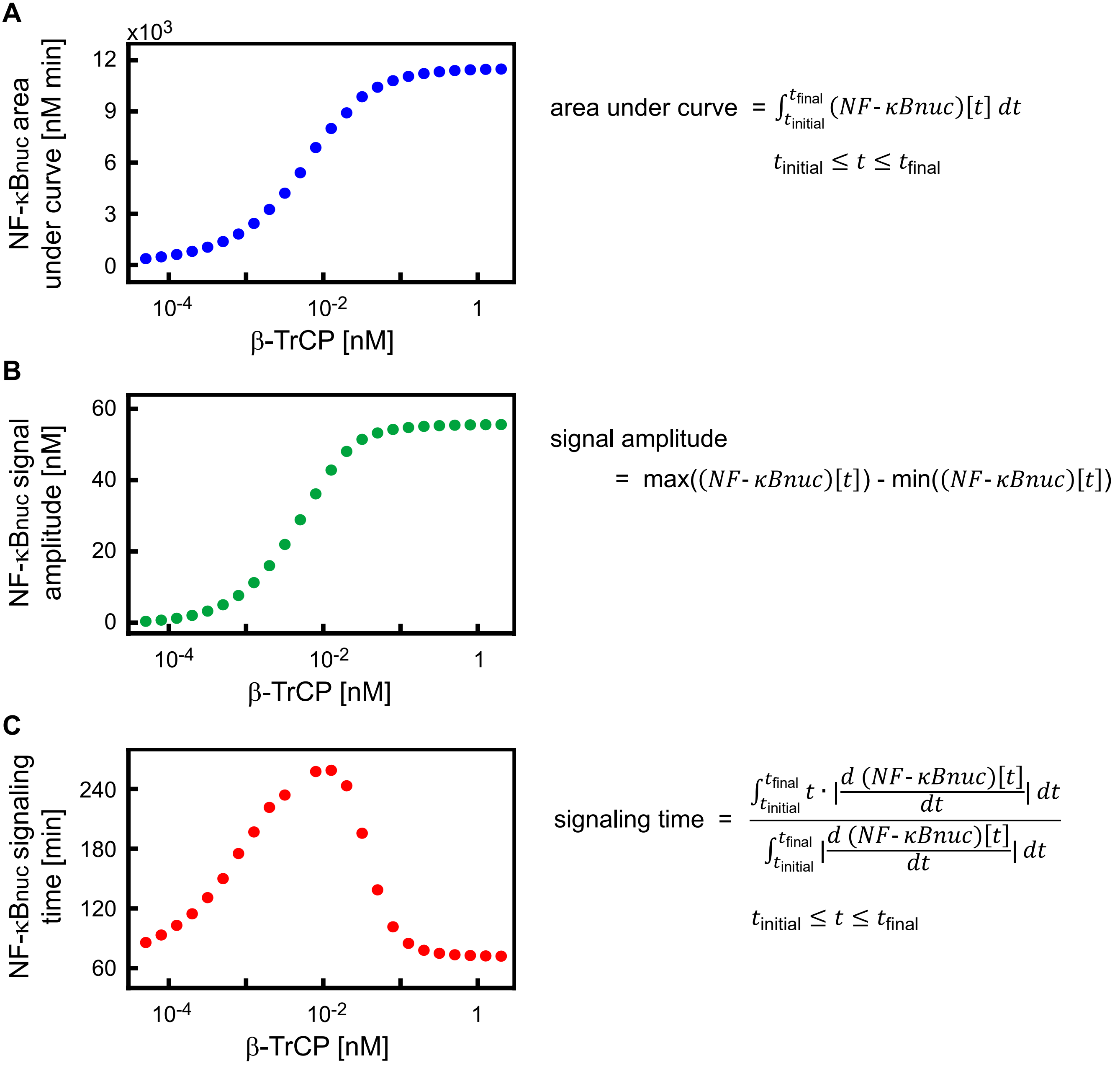

Figure S1: Influence of β-TrCP abundance on additional established measures of the transient dynamics of nuclear NF-κB upon TNF stimulation.

Dependence of the area under curve (A), signal amplitude (B) and signaling time (C) of nuclear NF-κB upon TNF stimulation assuming β-TrCP concentrations between $5 \cdot 10^{-5}$ nM and 2 nM. The respective definitions of the measures are shown on the right side of each panel. Signal amplitude can be interpreted as a measure of the magnitude of NF-κB activation, while signaling time characterises temporal aspects of the NF-κB response to TNF stimulation. The area under curve is a combination of magnitude and temporal aspects; it is referred to as time-integrated activity or accumulative response.
