# Supplementary material for: Controlling Nuclear NF-κB Dynamics by β-TrCP—Insights from a Computational Model
Source: Biomedicines. 2019 May 27;7(2):40. doi: 10.3390/biomedicines7020040 (PMC6631534; doi:10.3390/biomedicines7020040)
Supplement: Supplementary file 1 [file biomedicines-07-00040-s001.zip › Figure_S2.pdf]

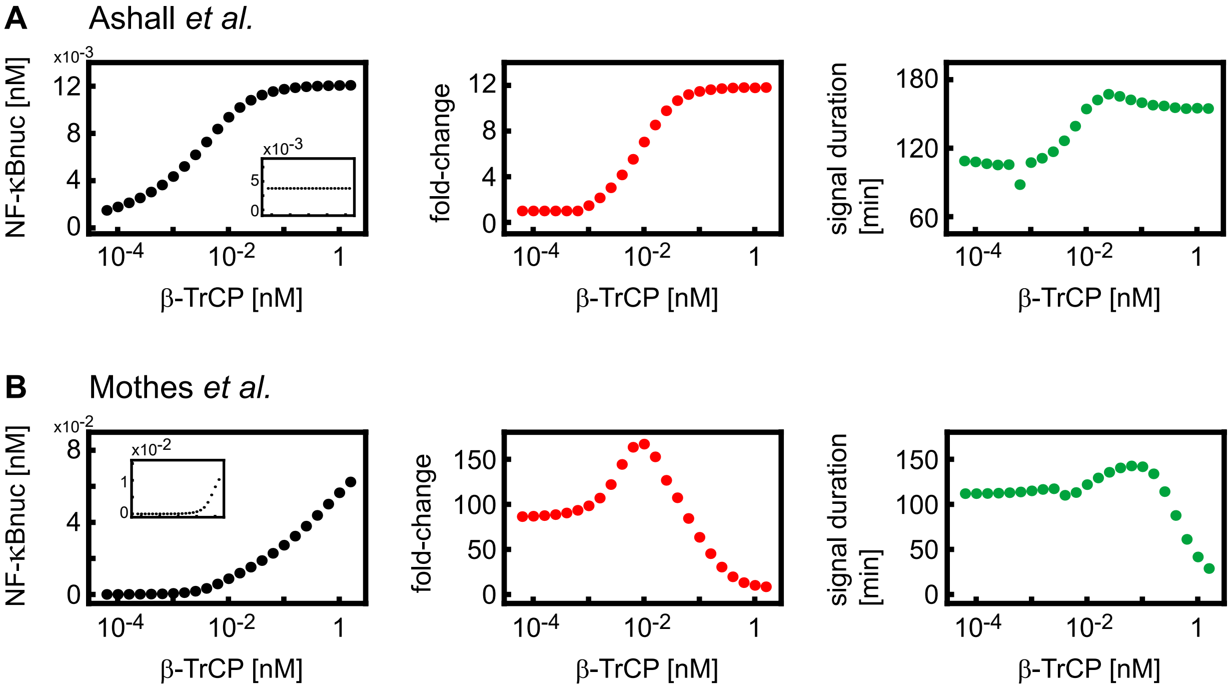

Figure S2: Confirmation of results in two other NF-κB pathway models.
The NF-κB pathway models published by Ashall et al. [2] and Mothes et al. [3] differ in their model structure and parametrization from the model of Lipniacki et al. [1] and thus also from our model. Neither Ashall et al. nor Mothes et al. include the pathway component β-TrCP. We integrate β-TrCP in the Ashall model by multiplying the parameters kt1a and kt2a each by β-TrCP. Similarly, we incorporate β-TrCP in the Mothes model by multiplying the parameter r4 by β-TrCP.
In both extended models, Ashall (A) and Mothes (B), we observe an increase of the steady state concentration of nuclear NF-κB after TNF stimulation with increasing β-TrCP concentrations (left panel in A and B). This finding is in agreement with the results of our model (Figure 3A). The small inlets show the influence of increasing β-TrCP concentrations on the steady state concentration of nuclear NF-κB without TNF stimulation.
Increasing β-TrCP concentrations also increase the fold-change of nuclear NF-κB in the extended Ashall model (middle panel in A). This result confirms the observations in our model (Figure 2D). The fold-change in the Mothes model also increases with β-TrCP concentrations until 10^{-2} nM β-TrCP (middle panel in B). Higher β-TrCP concentrations reduce the fold-change again. The Mothes model shows this biphasic relationship because changes in β-TrCP concentrations affect in addition the steady state concentration of nuclear NF-κB without TNF stimulation (small inlet in left panel in B). This is not the case in the Ashall model (small inlet in left panel in A) and our model (not shown).
Both extended models, Ashall and Mothes, show a biphasic relationship of signal duration and β-TrCP concentrations (right panel in A and B), as shown for the Lipniacki model (Figure 2E).
Taken together, the analyses of both extended models (Ashall and Mothes) demonstrate the robustness of the results found in the analysis of our model: that is, modulation of β-TrCP affects the steady-state concentration and the transient dynamics of nuclear NF-κB upon TNF stimulation.

References
[1] Lipniacki, T., et al., Mathematical model of NF-kappaB regulatory module. J Theor Biol, 2004. 228(2): p. 195-215.
[2] Ashall, L., et al., Pulsatile stimulation determines timing and specificity of NF-kappaB-dependent transcription. Science, 2009. 324(5924): p. 242-6.
[3] Mothes, J., et al., Sources of dynamic variability in NF-κB signal transduction: A mechanistic model. BioEssays : news and reviews in molecular, cellular and developmental biology, 2015. 37(4): p. 452-462.
