# Supplementary material for: Controlling Nuclear NF-κB Dynamics by β-TrCP—Insights from a Computational Model
Source: Biomedicines. 2019 May 27;7(2):40. doi: 10.3390/biomedicines7020040 (PMC6631534; doi:10.3390/biomedicines7020040)
Supplement: Supplementary file 1 [file biomedicines-07-00040-s001.zip › Figure_S3.pdf]

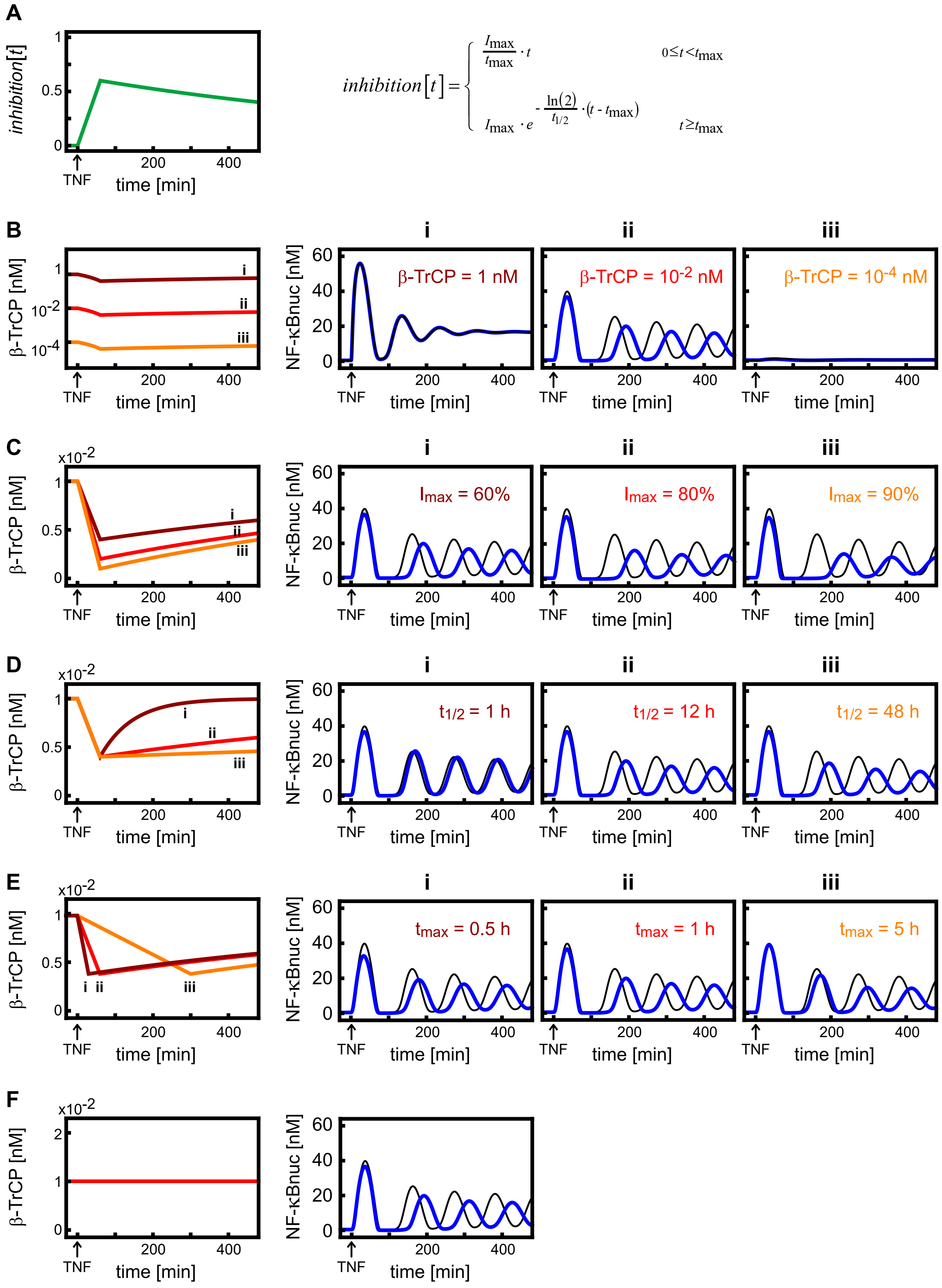

A
inhibition[t]
inhibition[t] = Imax/tmax · t   0≤t<tmax
Imax · e^(-ln(2)/t1/2 · (t - tmax))   t≥tmax
TNF
time [min]
B
β-TrCP [nM]
NF-κBnuc [nM]
i
ii
iii
β-TrCP = 1 nM
β-TrCP = 10-2 nM
β-TrCP = 10-4 nM
C
x10-2
Imax = 60%
Imax = 80%
Imax = 90%
D
t1/2 = 1 h
t1/2 = 12 h
t1/2 = 48 h
E
tmax = 0.5 h
tmax = 1 h
tmax = 5 h
F

Figure S3: Influence of a kinetic profile of a drug targeting β-TrCP on nuclear NF-κB dynamics. (A) To describe the kinetic profile of the mode of action of a drug, we use the stereotypical kinetic of a transient inhibition effect from the model of Sung et al. [1]. Their model is parameterized based on pharmacodynamics data of Bortezomib with a maximal inhibition (I_{max}) of 60% at time point (t_{max}) of 1h, after which inhibition decays with a half-life ($t_{1/2}$) of 12h. The kinetic profile using these particular parameters is shown.

(B-E) We assume the drug to inhibit the availability of β-TrCP for IκB binding, e.g. by acting as a competitive substrate. Following the approach of Sung et al., we substitute β-TrCP in all equations of our model by $[\beta\text{-}TrCP]\cdot(1-inhibition[t])$.

(B) *Inhibition*[*t*] is parameterized as in (A), which consequently reduces β-TrCP to the minimal level of 60% of its initial concentration at 60 min. Trajectories for the three different β-TrCP concentrations of Figure 2A-C are shown: (i) 1 nM, (ii) 10^{-2} nM, and (iii) 10^{-4} nM β-TrCP. The corresponding dynamics of nuclear NF-κB upon TNF stimulation are shown in the panels (i)-(iii). The black lines show the response dynamics in the absence of the drug and thus are identical to Figure 2A-C. The blue lines show the response dynamics in the presence of the drug. Note that in panels (i) and (iii) the black and blue lines are hardly distinguishable. The simulations demonstrate that a drug can change the response dynamics of nuclear NF-κB upon TNF stimulation at intermediate β-TrCP concentrations.

(C) Variation of maximal inhibition (I_{max}), assuming (i) 60%, (ii) 80%, (iii) 90%. β-TrCP concentration is 10^{-2} nM. Increasing I_{max} delays the second NF-κB peak and reduces peak amplitudes.

(D) Variation of half-life of inhibition ($t_{1/2}$), assuming (i) 1 h, (ii) 12 h, (iii) 48 h. β-TrCP concentration is 10^{-2} nM. NF-κB dynamics hardly differ in the presence or absence of the drug for short half-life (i). Longer half-life (e.g. 12h and 48h) have an impact on the NF-κB dynamics (ii, iii).

(E) Variation of time of maximal inhibition (t_{max}), assuming (i) 0.5 h, (ii) 1 h, (iii) 5 h. β-TrCP concentration is 10^{-2} nM. The later maximal inhibition is reached the later NF-κB dynamics differ between the presence or absence of the drug. This can be best observed at the first peak of NF-κB dynamics.

(F) Here we presume the drug to inhibit the activity of β-TrCP by reducing the rate of binding to IκB. In this case, we substitute the rate constants k8 and k10 by $k_8\cdot(1-inhibition[t])$ and $k_{10}\cdot(1-inhibition[t])$, respectively. *Inhibition*[*t*] is again parameterized as in (A). The simulation shows that the drug does not affect the concentration of β-TrCP (10^{-2} nM, left panel). The corresponding dynamics of nuclear NF-κB upon TNF stimulation (right panel) is changed in the presence of the drug (blue line) compared with its absence (black line). Note that the dynamics of nuclear NF-κB is identical to that in panel B(ii). The NF-κB dynamics (B(ii), F) are identical because the rate constants k8 and k10 form a product with β-TrCP in each relevant model equation such that multiplying the rate constants with (1- *Inhibition*[*t*]) is identical to multiplying β-TrCP with this term. The simulations demonstrate that the influence of the drug on nuclear NF-κB dynamics is independent of the drug's way of action (reduction of β-TrCP's abundance or inhibition of its binding activity). Importantly, a drug that inhibits β-TrCP's IκB binding activity does not change β-TrCP's abundance and may consequently preclude effects on other β-TrCP substrates. The small molecule inhibitor (GS143) may represent an example of such a drug.

References

1. Sung, M.H.; Simon, R. In silico simulation of inhibitor drug effects on nuclear factor-kappaB pathway dynamics. *Mol Pharmacol* **2004**, *66*, 70-75, doi:10.1124/mol.66.1.70 66/1/70 [pii].
